# System dynamics modeling of lake water management under climate change

**DOI:** 10.1038/s41598-022-09212-x

**Published:** 2022-04-06

**Authors:** Omid Bozorg-Haddad, Paniz Dehghan, Babak Zolghadr-Asli, Vijay P. Singh, Xuefeng Chu, Hugo A. Loáiciga

**Affiliations:** 1 Department of Irrigation and Reclamation Engineering, Faculty of Agricultural Engineering and Technology, College of Agriculture and Natural Resources, University of Tehran, Karaj, Tehran, Iran; 2 Department of Biological and Agricultural Engineering and Zachry Department of Civil and Environmental Engineering, Texas A&M University, 321 Scoates Hall, 2117 TAMU, College Station, TX 77843-2117 USA; 3 Department of Civil and Environmental Engineering, North Dakota State University, Dept 2470, Fargo, ND 58108-6050 USA; 4 Department of Geography, University of California, Santa Barbara, Isla Vista, CA 93106 USA

**Keywords:** Climate sciences, Ecology, Environmental sciences, Environmental social sciences, Hydrology, Natural hazards, Solid Earth sciences, Engineering, Mathematics and computing

## Abstract

Lake Urmia, the twentieth largest lake in the world, is the most valuable aquatic ecosystem in Iran. The lake water level has decreased in recent years due to human activities and climate change. Several studies have highlighted the significant roles of climatic and anthropogenic factors on the shrinkage of the lake. Management policies for water resources harvesting must be adopted to adapt to climate change and avoid the consequent problems stemming from the drought affecting Lake Urmia, and rationing must be applied to the upstream water demands. This study analyzes strategies and evaluates their effectiveness in overcoming the Urmia Lake crisis. Specifically, system dynamics analysis was performed for simulating the water volume of Lake Urmia, and the Hadley Centre coupled model was applied to project surface temperature and precipitation for two future periods: 2021–2050 and 2051–2080. Six management scenarios were considered for decreasing the allocation of agricultural water demand corresponding to two options: (1) one-reservoir option (Bukan reservoir only), and (2) six-reservoir option. The net inflow of Urmia Lake was simulated for the two future periods with the IHACRES model and with artificial neural network models under the six management scenarios. The annual average volumes of Lake Urmia would be 30 × 10^9^ and 12 × 10^9^ m^3^ over the first and second future periods, respectively, without considering the management scenarios. The lake volumes would rise by about 50% and 75% for the first and second periods, respectively under the management scenarios that involve strict protective measures and elimination of the effect of all dams and their reservoirs. Implementing strict measures would increase the annual average lake volume to 21 × 10^9^ m^3^ in the second period; yet, this volume would be less than the long-term average and strategic volume. The human water use would be completely eliminated under Scenario 6. Nevertheless, Lake Urmia would experience a considerable loss of storage because of drought.

## Introduction

Iran features arid and semi-arid climates. It receives one-third of the global average precipitation and has relatively high temperature due to its geographic location. The precipitation decrease along with an increase in temperature shows that Iran has become drier in the last few decades^1–3^. Shakouri and Taheri^4^ reported that, according to the 2006 census, the last ten-year average population growth rate in Iran was 1.61%, and the population growth is projected to range between 2 and 2.5% over the next decade. As population grows, water so does water use. Precipitation reduction, temperature increase, and the rise in water consumption in the last few decades have caused the groundwater levels to fall sharply, and have dried up many wetlands and lakes in Iran^5^. Climate change has exacerbated these conditions in northwestern Iran^6–10^.

One of the impacts of climate change and water harvesting in Iran is the shrinkage of Lake Urmia, located in northwestern Iran. Lake Urmia was the second largest saline lake in the world after the Dead Sea. On account of the natural features and unique ecology of Lake Urmia it has been designated as a national park and a protected region since 1967, as a Ramsar site since 1975, and as a UNESCO Biosphere Reserve since 1977^11^. The islands in the lake have been the habitat of flamingos (more than 20,000 pairs) and 200 to 500 pairs of white pelicans. However, plant and animal species have endured severe conditions in recent years because of drying in the Lake Urmia basin, reduction of the lake’s water volume, and significant rise in its salinity^12^. Some islands in the lake have become connected to the land as a result of the lake shrinkage. The high salinity of the lake water has extinguished species such as Artemia^13^. In addition to the severe threat on the wildlife by the lake’s drying up salt storms have become more frequent and harmful^14^.

Several studies have been reported addressing multifaceted water issues in Urmia Lake. Delavar and Morid^15^ simulated water level fluctuations of Lake Urmia with different methods, such as lake water balance, multiple regression equation (MRE), and artificial neural networks (ANNs). Results showed that the ANN method with concomitant use of accumulative inflow, monthly rainfall, and monthly evaporation had the best accuracy and the least sensitivity in simulating lake’s water level variations. Hassanzadeh et al.^16^ applied a system dynamics (SD) approach for simulating the effect of surface inflow on the declining Lake Urmia water level. The latter authors concluded that because of drought, over-harvesting of water resources, and dam construction in the basin, the lake water level has declined, and a quarter of the lake area morphed into saline land. Their results showed that the construction of four study reservoirs and surface water over-exploitation with their respective shares of 25% and 65% were the main causes for the lake level reduction. Noury et al.^17^ simulated the fluctuations of Lake Urmia water level with support vector machine (SVM) and neural wavelet network (NWN). Results showed that the SVM model performed better than the NWN model. Zarghami and Rahmani^18^ examined restoration plans for Lake Urmia with the SD method to increase irrigation efficiency and reduced the acreage of irrigated land. Hosseini-Moghari et al.^19^ showed that the water use for anthropogenic purposes in the Lake Urmia basin, being the dominant factor for the Lake Urmia crisis, induced 39–43% of the lake water loss (about 8 km^3^ during 2003–2013), up to 90% of the groundwater loss, and 39–45% of the lake-inflow reduction. Bozorg-Haddad et al.^20^ demonstrated the accuracy of the SD method in simulating the Urmia Lake water balance and evaluated the impacts of the agricultural water demand supplied by surface water on the Lake Urmia water level.

The severe hydrological drought of Lake Urmia prompted Iran’s government to create a ten-year program, named “Urmia Lake Restoration Program (ULRP)” in 2013 with a total cost of five billion US dollars^21^. The ULRP committee has entertained the potential of several alternative management scenarios to control the depletion of Urmia Lake^22^. As such, the following was proposed in the ULRP: reducing and controlling the withdrawal of surface water-groundwater resources in the basin, reducing the water consumption in the agricultural sector by 40%, and transporting water from the Zab and Aras Rivers and from the Caspian Sea^23^. Although numerous studies applied a wide range of methods to show the effects of the factors governing Lake Urmia’s level, none of them evaluated the relationship between the allocated water to the agricultural sector from the main reservoirs in the basin (under ULRP management scenarios) and the lake level.

This work’s objective is to evaluate management scenarios related to the ULRP to overcome the Lake Urmia crisis in two future periods, namely, 2021–2050 and 2051–2080, under climate change. Since climate change is a serious factor in the present pressing conditions of the Urmia Lake, the results of this research are more practical in comparison to previous studies. The SD method coded in the Vensim software is applied to simulate the current and future water balance of Urmia Lake under climate change. Mitigation strategies proposed in the ULRP are assessed under six management scenarios based on the modeling results for the future periods. This study aims to identify effective restoration measures for the policymakers to resolve the Lake Urmia crisis under climate change.

## Methodology

### System dynamics method

The SD method applies systemic processing to simulate complex non-linear dynamics and feedback. Systemic processing resorts to various tools to simulate complex system behavior and performance^24^. Systems evolve through states, which change with flows. An example of a state variable is water storage in the study of lakes. The SD method simulates changes in system states driven by flows and various feedbacks^25^.

This work employs the SD method to simulate storage change in Lake Urmia in one historical period (1957–2005) and two future periods (2021–2050 and 2051–2080). The lake’s water volume is the state variable, which is governed by inflows (precipitation, surface water inflows, and groundwater inflows) and outflows (evaporation, leakage, and surface water outflows). The lake’s mass balance equation is expressed as:

1 $$S_{t + 1} = \int\limits_{t}^{t + 1} {[I_{s} - O_{s} ]ds + S_{t} }$$

where *S*_*t*+*1*_ , *S*_*t*_*, I*_*s*_, and *O*_*s*_ denote the lake’s storage at time *t* + *1*, the lake’s storage at time *t*, the inflow rate to the lake at time *s* (units of volume/time), and the outflow rate from the lake at time *s* (units of volume/time)*,* respectively.

The SD method employs the Euler and Runge Kutta methods for the solution of differential equations. The software STELLA, Vensim, Powersim, and Dynamo feature SD solvers^26^. This work applies the widely-used Vensim software^27^.

### Climate change

The data sets needed for modeling Lake Urmia’s storage over the two future periods were generated after simulating the lake’s water balance during the historical period. HADCM3, a coupled atmosphere–ocean general circulation model’s (AOGCM) climate projections were used to generate precipitation and surface temperature projections over the future periods. The AOGCM data at coarse spatial scales were downscaled to the regional scale suitable for lake storage simulation. The commonly used downscaling methods are statistic and dynamic in nature^28,29^. This works applies the delta-change downscaling method, in which monthly temperature and precipitation differences between the future and historical are calculated by^29^:

2 $$\Delta T_{t} = \overline{T}_{GCM,fut,t} - \overline{T}_{GCM,hist,t}$$

3 $$\Delta P_{t} = \overline{P}_{GCM,fut,t} - \overline{P}_{GCM,hist,t}$$

where *∆T*_*t*_ denotes the difference in long-term average temperatures simulated by HADCM3 for the future ($\overline{T}_{GCM,fut,t}$) and historical ($\overline{T}_{GCM,hist,t}$) periods in month *t* (°C); *∆P*_*t*_ represents the difference in long-term average precipitations simulated by HADCM3 for the future ($\overline{P}_{GCM,fut,t}$) and historical ($\overline{P}_{GCM,hist,t}$) periods in month *t* (mm). Then, *∆T*_*t*_ and *∆P*_*t*_ are applied to project the future downscaled data as follows^29^:

4 $$T_{t} = T_{obs,t} + \, \Delta T_{t}$$

5 $$P_{t} = P_{obs,t} { + }\Delta P_{t}$$

where *T*_*obs,t,*_ and *P*_*obs,t*_ denote respectively the observed temperature (°C) and precipitation (mm) in month *t* in the baseline period; and *T*_*t*_ and *P*_*t*_ are the downscaled temperature (°C) and precipitation (mm) in month *t* of the future period, respectively. Delta-change downscaling is a simple yet efficient option when it comes to spatial downscaling of climate change projections (e.g.^30–32^). The gist of this method is to replicate the changing patterns that are projected by the atmospheric ocean general circulation models (AOGCMs) to generate the climate change patterns of hydro-climatic variables on a regional scale. As such, one would simply compute the relative changes in the long-term variations of the variable that is projected by the models within the baseline and future timeframes. These relative changing patterns would be applied to the historical data to project the impact of climate change on a local scale.

### Rainfall-runoff modeling

The IHACRES (identification of unit hydrographs and component flows from rainfall, evapotranspiration and streamflow) model is herein applied to simulate runoff from precipitation. Ashofteh et al.^33^ implemented the IHACRES model to investigate the effects of climate change on reservoir performance in agricultural water supply. Ashofteh et al.^34^ evaluated the probability of flood occurrence in future periods with IHACRES.

The IHACRES model includes a non-linear loss module and a linear unit hydrograph module. The non-linear loss module converts the observed rainfall into the effective rainfall, after which the linear unit hydrograph module converts the effective rainfall into the simulated streamflow^35^. Here, precipitation *r*_*k*_ in time step *k* is converted to effective precipitation *u*_*k*_ through the non-linear loss module employing a catchment wetness index *s*_*k*_*:*

6 $$u_{k} = \, s_{k} \times \, r_{k}$$

The effective precipitation is converted to the surface runoff in time step *k* with the linear unit hydrograph module. The parameters of this model can be set through a thorough grid numeric search and trial-and-error. Perhaps, one of the major advantages of the IHACRES model over other commonly-used rainfall-runoff models is its minimal input data requirement (i.e., air temperature and precipitation)^31,35^.

The other alternative for hydrologic simulation is to use data-driven models. Here, the multilayer perceptron (MLP), a variety of the artificial neural network (ANN) method, was also used to simulate runoff. This model consists of an inlet layer, one or several middle (hidden) layer(s), and an output layer. All of the neurons of a layer are connected to the ones in the next layer, forming a network with complete connections. The primary parameters in modeling the neural network of MLP are: (1) the number of neurons in each layer, (2) the number of layers in the network, and (3) the forcing functions. A regular MLP neural network has three layers^36^. The first and the third layers are respectively the system inputs and outputs. The middle layer consists of neurons that perform calculations on the inputs. Choosing the number of layers in a neural network is made by trial and error^37^. From a hydrological simulation standpoint the main idea behind this model is to create a suitable artificial neural network that is capable of accurately converting a set of hydro-climatic variables such as precipitation and temperature as input data into streamflow values. It should be noted that, like most data-driven models, the process of opting for a proper neural network architecture (i.e., selecting the number of layers, number of neurons, and the forcing function) is, for the most part, a trial-and-error procedure.

One must objectively evaluate the performance of the hydrological models in order to opt for the setting of a suitable parameter. The root mean square error (*RMSE*), coefficient of determination (*R*^2^), and mean absolute error (*MAE*) are herein employed to assess the performance of the rainfall-runoff model. They are respectively calculated as follows:

7 $$RMSE = \sqrt {\frac{{\sum\limits_{t = 1}^{N} {(x_{t} - y_{t} )^{2} } }}{N}}$$

8 $$R^{2} = \left( {\frac{{\sum\nolimits_{t = 1}^{N} {(x_{t} - \overline{x} ).(y_{t} - \overline{y} )} }}{{\sqrt {\sum\nolimits_{t = 1}^{N} {(x_{t} - \overline{x} )^{2} } } .\sqrt {\sum\nolimits_{t = 1}^{N} {(y_{t} - \overline{y} )^{2} } } }}} \right)^{2}$$

9 $$MAE = \frac{{\sum\nolimits_{t = 1}^{N} {\left| {x_{t} - y_{t} } \right|} }}{N}$$

where *x*_*t*_ , *y*_*t*_*,* and *N* denote the simulated value in time step *t*; the observed value in time step *t;* and the number data values, respectively. Large errors have a disproportionately large effect on *RMSE* or *MAE*.

### Performance criteria

Various quantitative measures can be used to assess the performance of water resources systems under different strategies. When it comes to water resources planning and management, perhaps, some of the most common performance criteria are the probability-based performance criteria (PBPC) (i.e., reliability, vulnerability, and resiliency)^31,38^. In this context, reliability represents the probability of successful functioning of a system; resiliency measures the probability of successful functioning following a system failure; lastly, vulnerability is the severity of failure during an operation horizon^39,40^. The basic idea behind a performance evaluation attribute is to provide a quantitative measure to describe and assess the performance of a system. In the context of water resources planning and management, these measures have proven time and again that they can be reliable options to evaluate a set of strategic management options objectively (see, e.g.^40–43^, and^44^, just to name a few).

### Operating policy

Any water resources system requires something called the “rule curve,” which determines how water is allocated in a given situation^45^. A common and effective rule curve when it comes to operation of water resource systems is the standard operation policy (SOP). SOP is a simple, and perhaps best-known real-time operation policy in water resources planning and management^46^. The core principle here is to minimize the water shortage at the current time step with no conservation policy (e.g., hedging rules) in place. The SOP, as a standard rule curve, determines how the operator acts to control a system at any given state of a reservoir^47,48^. This rule curve is established as an attempt to balance various water demands including but not limited to flood control, hydropower, water supply, and recreation^49^. A SOP operating system attempts to release water to meet a water demand at the current time, with no regard to the future. Thus, according to the SOP’s principle, the decision-makers, first allocate the available water to meet the demand of the stakeholder with the highest priority. After this first water demand is fully satisfied, the available water can be used for the next demand. Such an allocation process continues until no water is available.

### Ethics approval

All authors accept all ethical approvals.

### Consent to participate

All authors consent to participate.

### Consent to publish

All authors consent to publish.

## Case study

**Figure 1:**
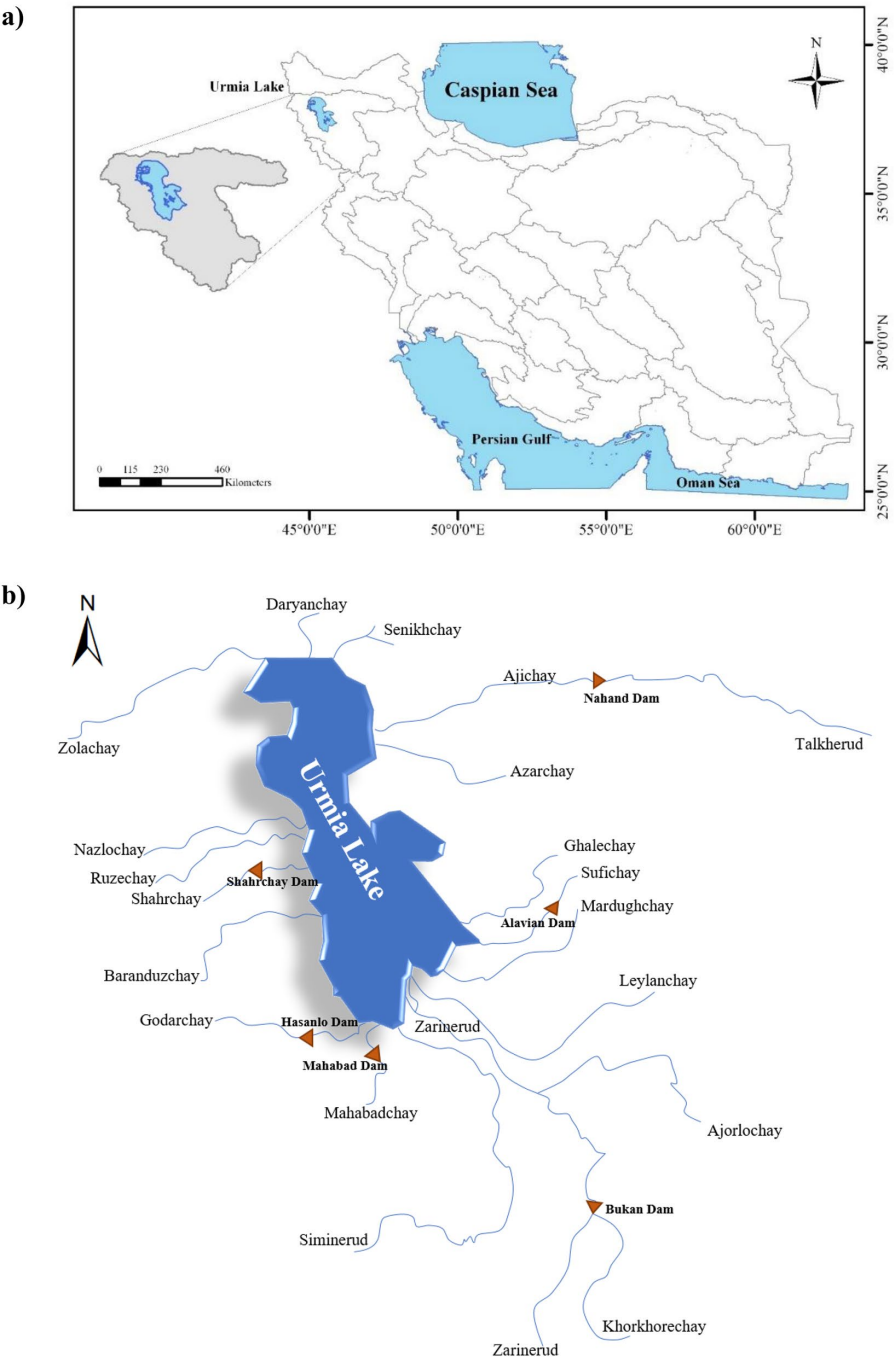
Case Study (**a**) Location of the Lake Urmia basin; (**b**) Schematic of the river drainage network in the Lake Urmia basin. Not drawn to scale.

The Lake Urmia basin with an area of 51,862 km^2^ is located within 35° 40ʹ and 38° 30 ʹ N (latitude) and within 44° 07 ʹ and 47° 53 ʹ E (longitude), as shown in Fig. 1 (part a). Urmia Lake is the largest lake with the richest aquatic ecosystem in Iran. The lake is located in the northwestern region of Iran between West and East Azarbaijan provinces. The average annual precipitation varies from 250 mm in the lowlands to 1,000 mm in the western regions of the basin. The dominant climate Mediterranean with low precipitation during the summer. The Lake Urmia basin is a typical endorheic (or closed) basin, in which all surface water and groundwater drain into the lake.

**Figure 2:**
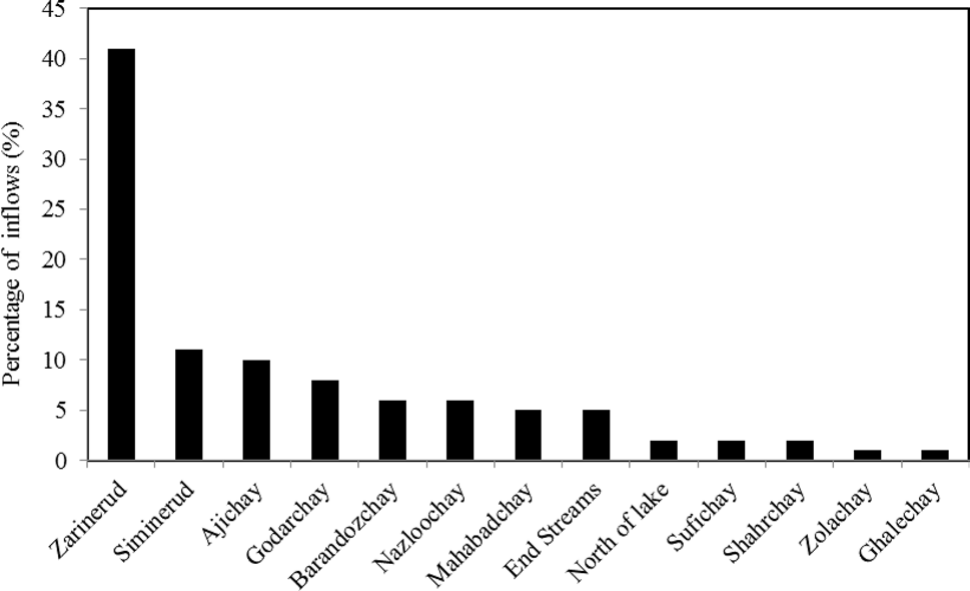
Contribution percentages of Lake Urmia water inflows.

The Zarinerud and Siminerud Rivers are the two largest rivers in the basin and account for about 52% of the total surface water inflow of Lake Urmia. Figure 1 (part b) depicts a schematic of the river drainage network of the Lake Urmia basin. Figure 2 displays the percentages of runoff contributions from all major tributaries of Lake Urmia.

There are seven large active reservoirs (i.e., Bukan, Mahabad, Alavian, Shahrchai, Hasanlou, Nahand, and Sarough), seven under-construction reservoirs, and 10 under-study reservoirs in the Lake Urmia basin. The Sarough reservoir was not considered in this study because it was recently constructed and there are no operational data prior to 2012. Tables 1 and 2 respectively list the characteristics of the six operational reservoirs in the Lake Urmia basin and their inflows, water demands, and water supplies. The six management scenarios under the climate change condition and their control volumes of the lake’s water are summarized in Table 3. The said values are there to show, based on different conditions that are depicted in the said table, how much one should reduce the water demand for the agriculture sector, which by far is the most dominant water-demanding sector in the region. It should be noted that the stated conditions are based on the lake’s volume in a given month and its relative status to the long-term average of the lake volume and the lake ecological volume in the said month. The general idea being that the more water stresses are imposed on the lake, the more pronounced restriction must be applied to the demand to control and potentially restore the water of the Urmia Lake. The water allocated to the agricultural sector from either the Bukan reservoir (Option 1) or all six reservoirs (Option 2) were determined under each scenario based on the simulation of lake storage. This study has chosen the SOP as the operating policy. Based on the Urmia Lake Restoration Program (ULRP) recommendations these priorities, from the highest to the lowest one, are drinking, ecological, agricultural, and industrial demands.

**Table 1:** Characteristics of operational dams/reservoirs in the Urmia Lake basin (adopted from^23^).

Specification	Unit	Reservoirs					
		Mahabad	Bukan	Alavian	Nahand	Hasanlou	Sharchay
Operation year	–	1970	1971	1995	1996	2000	2004
Design volume	10^6^ m^3^	230	762	60	24	101.27	221
Active volume	10^6^ m^3^	190	654.4	57	21.1	99	203
Annual adjustment volume	10^6^ m^3^	195	1159	123.4	32	93	119
Area under cultivation	Ha	18,200	59,369	12,195	0	16,500	12,500

**Table 2:** Inflows, water demands, and water supplies of the six reservoirs (adopted from^23^).

Dam/reservoir name	Inflow (10^6^ m^3^)	Water supply (10^6^ m^3^)	Water demand (10^6^ m^3^)
Bukan	1629	915	1199.3
Nahand	31	19	28
Mahabad	272	169	231
Hasanlou	93	27	38
Alavian	115	78	123
Shahrchai	187	127.7	179
	Total amount	1335.7	1798.3
	Deficit (10^6^ m^3^)		462.6

**Table 3:** Six management scenarios and their lake water control volumes (adopted from^22,23^).

Management scenarios	Lake water volume condition		
	V_ave_ < V_t_	V_eco_ < V_t_ < V_ave_	V_t_ < V_eco_
1	100	100	100
2	100	100	90
3	100	90	70
4	100	70	50
5	100	50	0
6	100	0	0

V_t_ = lake volume in month *t* (million m^3^); V_ave_ = long-term average of the lake volume in month *t* (million m^3^); and V_eco_ = lake ecological volume in month *t* (million m^3^) (i.e., the minimum lake water volume for the survival of living organisms in the Lake Urmia ecosystem).

## Results and discussion

### Simulation model for historical data

**Figure 3:**
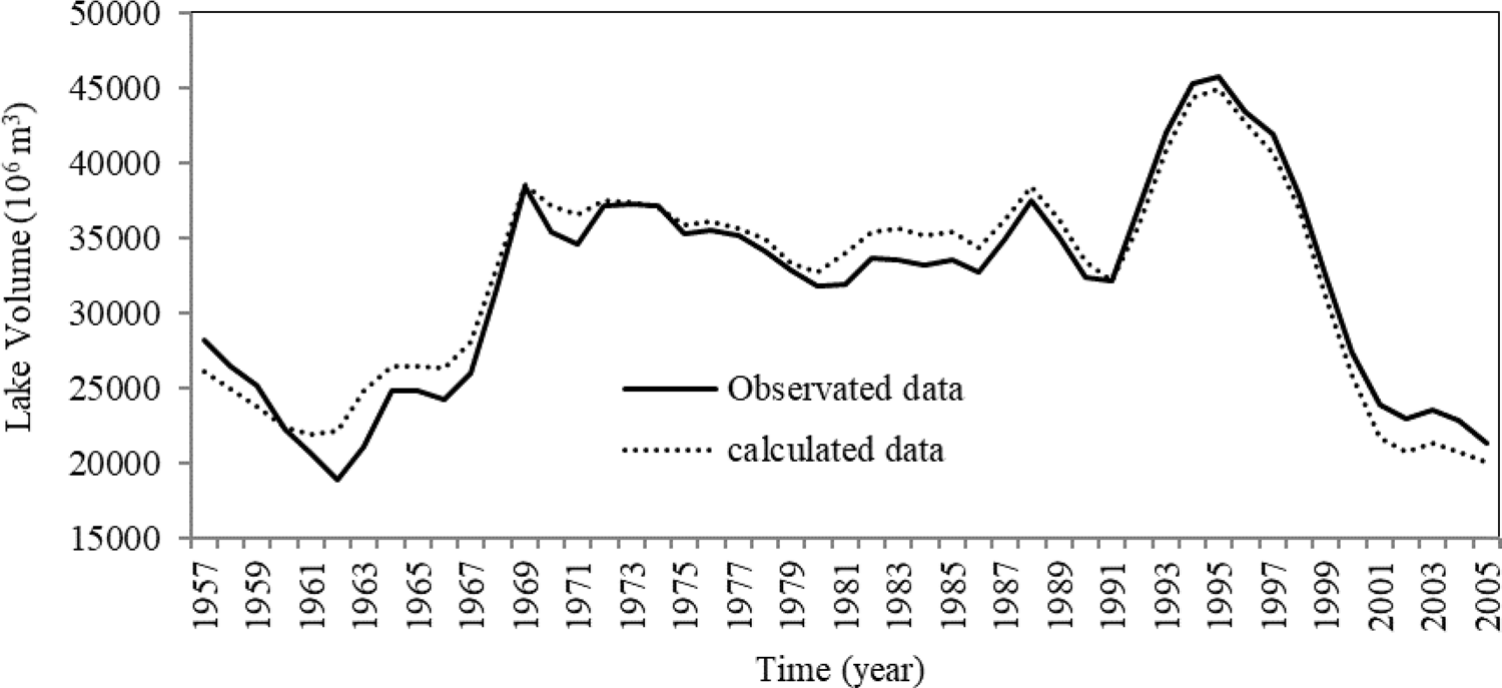
Comparison of the observed and calculated lake volumes.

**Figure 4:**
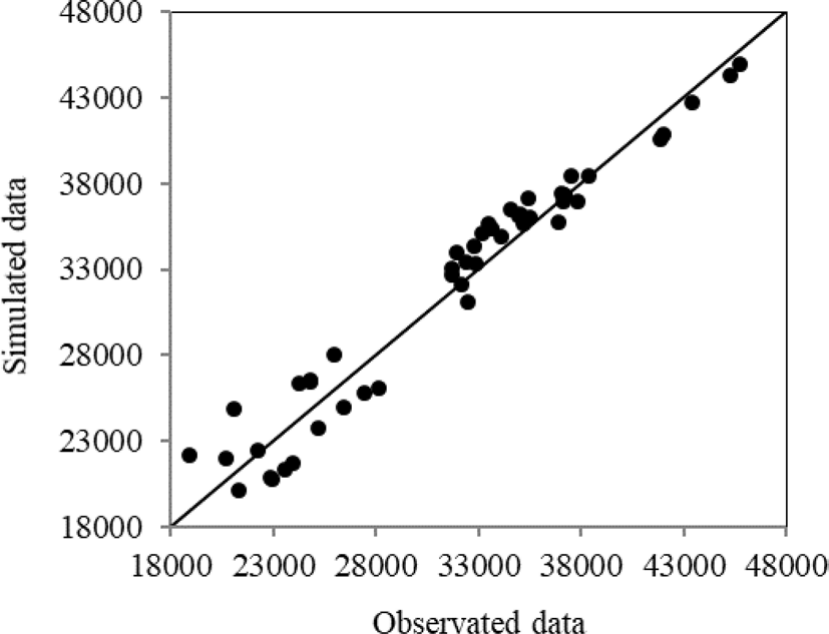
Scatter plot of the simulated and observed lake volumes.

The SD method performance in simulating the lake volume was evaluated by comparing simulated values with the observed data. Figure 3 shows the comparison of the observed and simulated water volumes of Urmia Lake in 1957–2005. The values of *R*^2^ and *RMSE* were 0.952 and 18.2 × 10^6^ m^3^, respectively. Figure 4 depicts a comparison between the simulated and observed lake water volumes, indicating that most data points are scattered near the 1:1 (45°) line, although the maximum simulated lake volume was slightly smaller than the observed one. It is concluded that the overall performance of the SD method was acceptable based on the calculated results.

### Downscaling results

**Figure 5:**
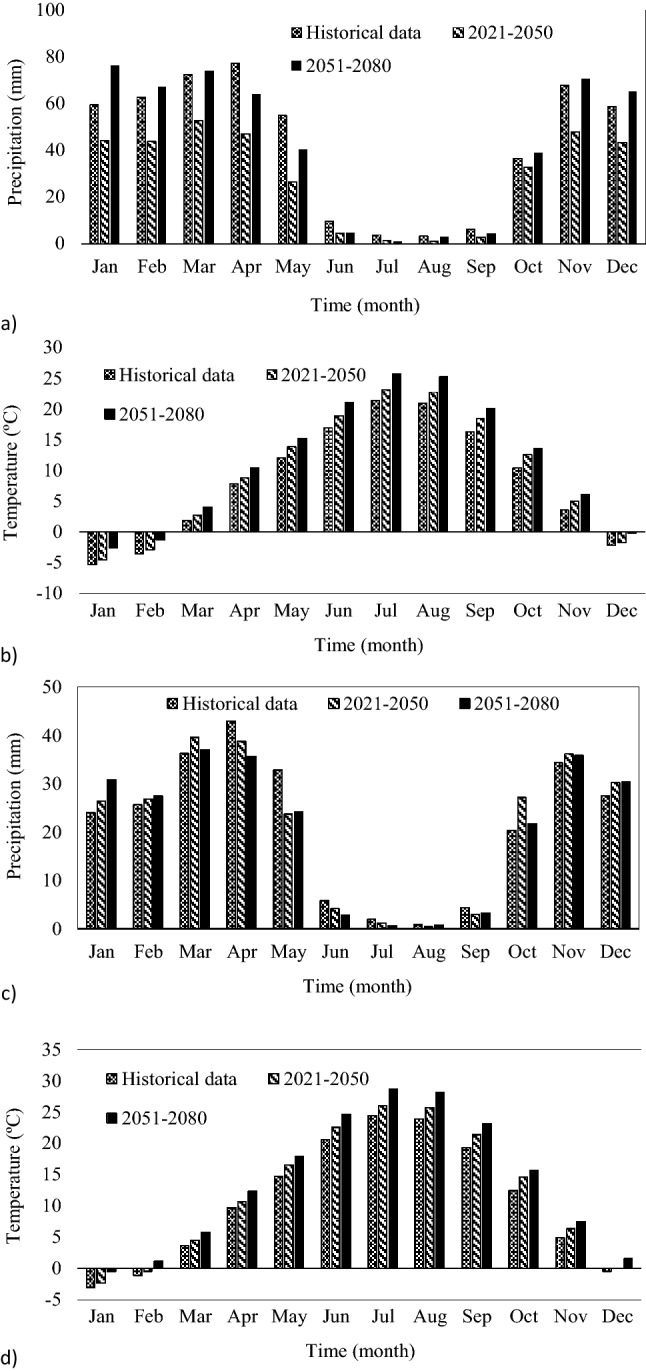
Average monthly precipitation and temperature in the Bukan sub-basin corresponding to the historical period and two future periods: (**a**) precipitation in the upstream region; (**b**) temperature in upstream region; (**c**) precipitation in the downstream region; (d) temperature in the downstream region.

The AOGCM projections were downscaled to the local scale. The temperature and precipitation projections were extracted and then simulated by the HADCM3 under the A2 emission scenario corresponding to the study region. Downscaling was performed with the delta-change method for 2021–2050 and 2051–2080. Figure 5 displays the downscaled data corresponding to the regions downstream and upstream Bukan reservoir in the two future periods. Results demonstrated that the temperature would increase in the Bukan basin in both future periods. The increase in the predicted temperatures corresponding to the upstream and the downstream regions range between 1.38 and 3.08 °C in 2021–2050, and between 1.39 and 3.1 °C in 2051–2080, respectively. According to Fig. 5a it is expected to have a 32% reduction in precipitation in the region upstream of Bukan reservoir during 2021–2050, while the corresponding precipitation would increase by 0.4% during 2051–2080. The precipitation downstream of Bukan reservoir exhibits a rising trend of 0.3% in 2021–2050 and 2.38% in 2051–2080.

### Rainfall-Runoff modeling

The number of neurons between 1 and 20 was investigated in the ANN model. Here, precipitation and temperature were considered as the input variables. 12 neurons were chosen as the optimal number by trial and error. Sensitivity analysis was done on the activation function for the input and output layers. The results showed that the sigmoid function had a suitable performance as a non-linear activator function in the output layer. 70% of the data were used for calibration and the remaining 30% of the data were used for validation of the ANN and IHACREs models. The calibration and validation data included the monthly average temperatures and precipitations and the inflows into the Bukan reservoir in 1982–2002 and 2003–2011.

Results of the performance measures of the two models for calibration and validation are listed in Table 4. The ANN model performed better than IHACRES in terms of *R*^2^, *MAE*, and *RMSE*. In calibration the ANN model had a better performance with an increase of 13.5% in *R*^2^, and 11% and 33% decreases in *RMSE* and *MAE*, respectively, in comparison with the IHACRES model. In validation the ANN model had a 3% increase in *R*^2^, and 5% and 18% reductions in *MAE* and *RMSE,* respectively, in comparison with the IHACRES model. Therefore, the ANN model was implemented to simulate the inflow to the Bukan reservoir in the two future periods.

**Table 4:** Statistical metrics for the IHACRES and ANN models.

Model	Phase	*RMSE* (10^6^ m^3^)	*MAE* (10^6^ m^3^)	*R*^2^
IHACRES	Calibration	10.6	69.25	0.74
	Validation	8.27	46.19	0.82
ANN	Calibration	9.49	46.24	0.84
	Validation	7.81	37.57	0.85

**Figure 6:**
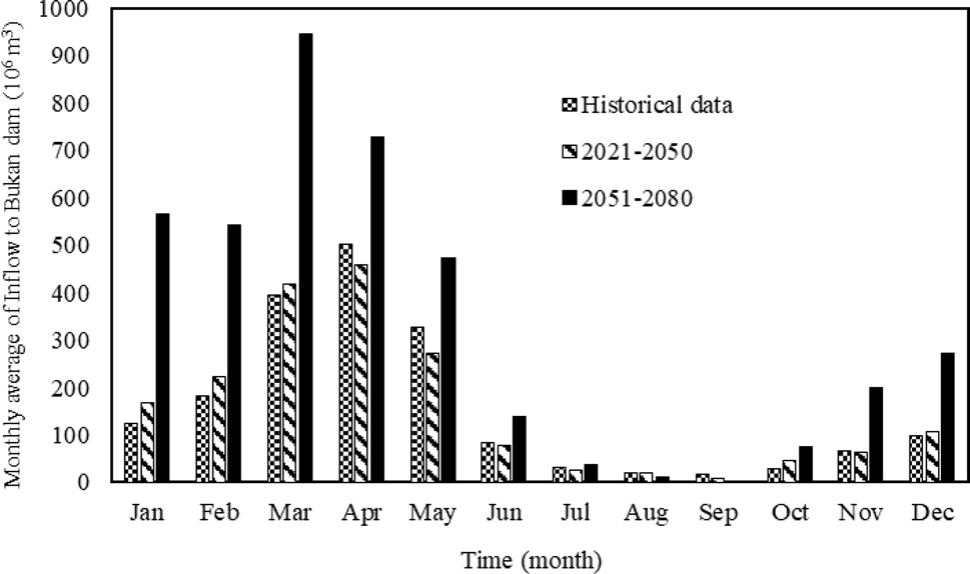
Simulated Bukan reservoir monthly inflows in the historical period and the two future periods.

Figure 6 shows the inflow to the Bukan reservoir would increase by 1.07 × 10^6^ m^3^ and 176.33 × 10^6^ m^3^ in the first and second future periods (compared to the historical data), respectively. According to Fig. 6 in the first future period the increase of inflow to the Bukan reservoir is evident from January through March, October, and December. However, the inflow would be reduced from April through July. The inflow changes would be more significant in the second future period than the first one. The largest inflow in the second future period would be in March, while the peak inflow in the historical period would be in April. It can be concluded that the inflow to the Bukan reservoir would change in the future.

### Reservoir water supplies in the future periods

**Figure 7:**
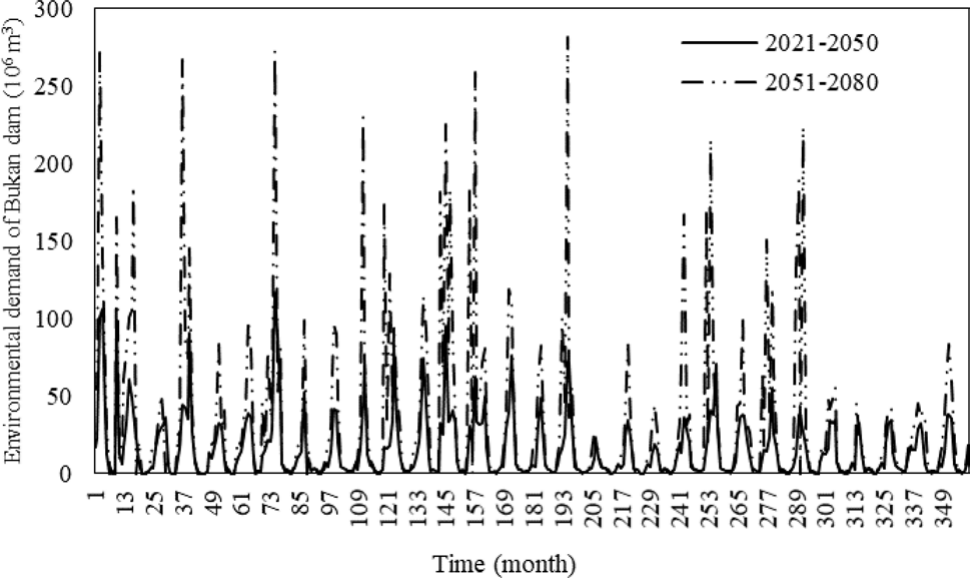
Environmental water demands of the Bukan reservoir in the two future periods.

The water supplies from the Bukan reservoir were calculated for the two future periods. Table 5 lists the monthly potable and industrial water demand, agricultural water demand, and Fig. 7 depicts the environmental water demand from the Bukan reservoir in the future periods^22,23^. Evapotranspiration in the agricultural sector and evaporation from the lake would increase due to the increased temperature in both future periods, and the potable and industrial water demand would increase due to the population growth. As expected, the agricultural, environmental, and potable and industrial water demands would increase. Also, the water inflow to the lake under climate change would increase, so the share of the lake from it would increase. However, the evaporation of the lake could be greater than the increased environmental water demand because of the temperature increment. The increase in the potable and industrial water demand would be negligible in comparison with the rise in the water demands of the agricultural and environment sectors.

**Table 5:** Monthly potable, industrial, and agricultural water demands from the Bukan reservoir in the two future periods (10^6^ m^3^) (adopted from^22,23^).

Sector	Periods	Jan	Feb	Mar	Apr	May	Jun	Jul	Aug	Sep	Oct	Nov	Dec
Potable and industrial	2021–2050	17.2	17.1	16.5	15.3	17.1	17.7	18.5	18.3	17.6	17.6	16.1	16.8
	2051–2080	17.4	17.3	16.7	15.5	17.3	18	18.7	18.6	17.9	17.9	16.3	17.0
Agricultural	2021–2050	0	0	13.2	96.8	298.1	492.2	558.1	475.2	313.2	122.1	0	0
	2051–2080	0	0	13.9	102	314.2	512.1	571.4	485.9	322.4	125.7	0	0

The potable and industrial demands in various months would be nearly constant (about 17 × 10^6^ m^3^ with a variation of 1 × 10^6^ m^3^). The agricultural water demand would zero in the four cold months of the year when the river inflow was much higher. Therefore, the Bukan reservoir stores water during the cold season for regulating water for agricultural use during the growth season. Among all water demands, the agriculture sector would have the highest water demand from June through August, and its average monthly value equals 500 × 10^6^ m^3^. Figure 7 shows considerable variations in the monthly lake’s environmental water demands, which are similar to the variations in the inflows of the lake. The environmental water demand for the lake in the second future period (2051–2080) would be larger than the first future period’s (2021–2050).

### Simulation of Lake Urmia water stage in the future periods

The variables for water allocation of a reservoir in this study include: reservoir inflow, flow downstream the reservoir, and water demands of the agricultural, environmental, potable, and industrial sectors. These variables for the Bukan reservoir were compared with those for the five other operational reservoirs in the Lake Urmia basin. Specifically, the ratios of these variables for the five reservoirs to the corresponding variables for the Bukan reservoir in the historical period were calculated (see Table 6). The ratios presented in Table 6 were used to calculate the inflow and outflows of the five other reservoirs in the future periods. The reservoir inflow and its downstream flow in the future periods were calculated only for the Bukan reservoir.

**Table 6:** Ratios of five variables of five reservoirs to the corresponding values for the Bukan reservoir in the historical period (partially adopted from^22,23^).

Reservoir name	Reservoir variables	Jan	Feb	Mar	Apr	May	Jun	Jul	Aug	Sep	Oct	Nov	Dec
Mahabad	IN	0.15	0.17	0.20	0.19	0.15	0.10	0.09	0.08	0.09	0.13	0.14	0.15
	AgD	0.02	0.02	2.32	1.51	0.82	0.34	0.18	0.19	0.17	0.16	0.11	0.08
	PID	0.08	0.08	0.08	0.09	0.08	0.07	0.07	0.07	0.07	0.07	0.08	0.08
	ED	0.20	0.84	2.70	3.32	1.55	0.10	0.03	0.02	0.01	0.03	0.09	0.15
	DF	0.07	0.15	0.20	0.17	0.15	0.44	1.32	1.71	1.21	0.36	0.13	0.06
Alavian	IN	0.05	0.04	0.04	0.06	0.10	0.13	0.24	0.20	0.22	0.14	0.07	0.05
	AgD	0.48	0.37	2.32	0.14	0.03	0.01	0.01	0.01	0.02	0.17	0.31	0.53
	PID	0.13	0.13	0.13	0.14	0.13	0.12	0.12	0.12	0.12	0.12	0.14	0.13
	ED	0.06	0.07	0.46	1.21	1.18	0.60	0.07	0.04	0.04	0.05	0.05	0.05
	DF	0.05	0.04	0.08	0.12	0.11	0.05	0.00	0.00	0.02	0.04	0.05	0.06
Nahand	IN	0.01	0.01	0.01	0.02	0.03	0.04	0.03	0.02	0.03	0.03	0.02	0.01
	PID	0.17	0.17	0.18	0.20	0.18	0.17	0.16	0.16	0.17	0.17	0.19	0.18
	ED	0.02	0.02	0.12	0.31	0.31	0.12	0.01	0.00	0.00	0.01	0.01	0.01
	DF	0.71	0.69	0.69	0.71	0.75	0.81	0.26	0.32	0.49	0.70	0.70	0.73
Hasanlou	IN	0.13	0.08	0.04	0.03	0.03	0.05	0.09	0.09	0.16	0.21	0.17	0.14
	AgD	0.12	0.12	0.21	0.17	0.14	0.12	0.12	0.11	0.09	0.09	0.01	0.12
	DF	0.01	0.04	0.30	0.68	1.22	2.12	0.73	0.02	0.00	0.01	0.02	0.03
Shahrchay	IN	0.04	0.02	0.03	0.07	0.19	0.54	0.60	0.32	0.18	0.12	0.08	0.05
	AgD	0.27	0.27	0.09	0.11	0.17	0.24	0.30	0.33	0.32	0.30	0.01	0.27
	PID	0.18	0.18	0.18	0.20	0.18	0.17	0.16	0.17	0.17	0.17	0.19	0.18
	ED	0.05	0.06	0.43	1.50	2.31	1.70	0.49	0.05	0.03	0.04	0.05	0.05
	DF	0.00	0.01	0.02	0.05	0.11	0.23	0.12	0.01	0.01	0.02	0.02	0.01

*IN* Inflow; *AgD* Agricultural water demand; *PID* Potable and industrial water demand; *ED* Environmental water demand; *DF* Downstream flow.

**Figure 8:**
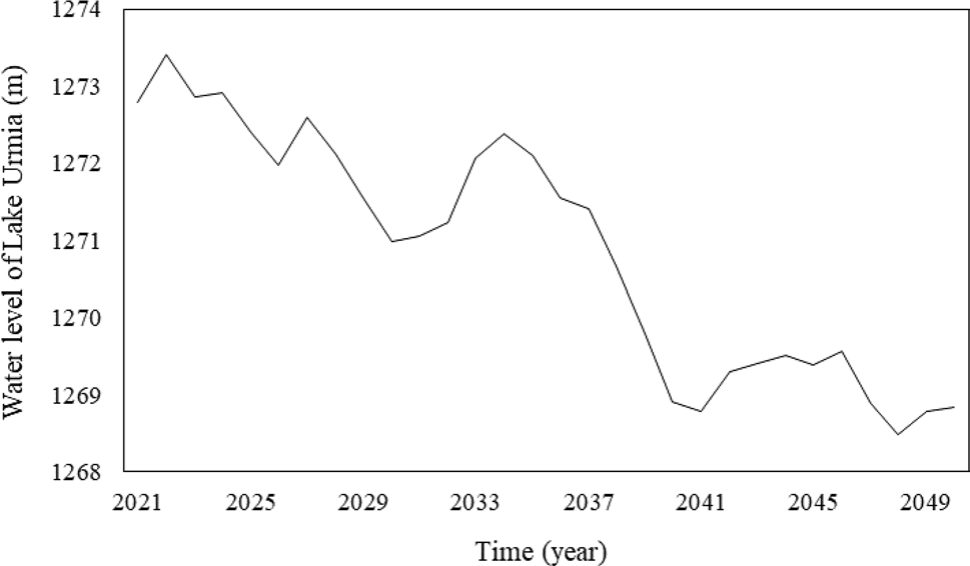
Predicted water levels of Lake Urmia in 2021–2050.

**Figure 9:**
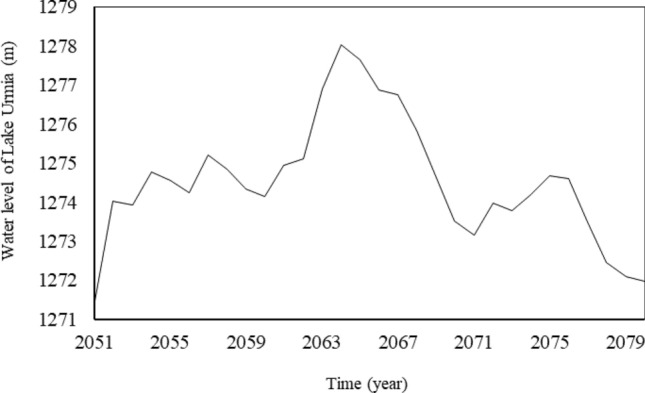
Predicted water levels of Lake Urmia in 2051–2080.

The simulated water stages of Lake Urmia in the first and second future periods are respectively shown in Figs. 8 and 9. The lake water level in the period 2021–2050 would be lower than its long-term average (1,275.86 m), but also lower than the ecological water level (i.e., the minimum water level for the survival of living organisms in the Lake Urmia ecosystem is 1,274.10 m). Remedial strategies have been proposed to prevent such a crisis in the basin in the Urmia Lake Restoration Program (ULRP). Specifically, management must be implemented to reduce the agricultural water use. Reducing agricultural water allocation is examined in the following section. In the second future period the lake water level would be slightly higher than the corresponding historical average water level determined by the mass balance analysis.

### Comparison of the proposed management scenarios

**Figure 10:**
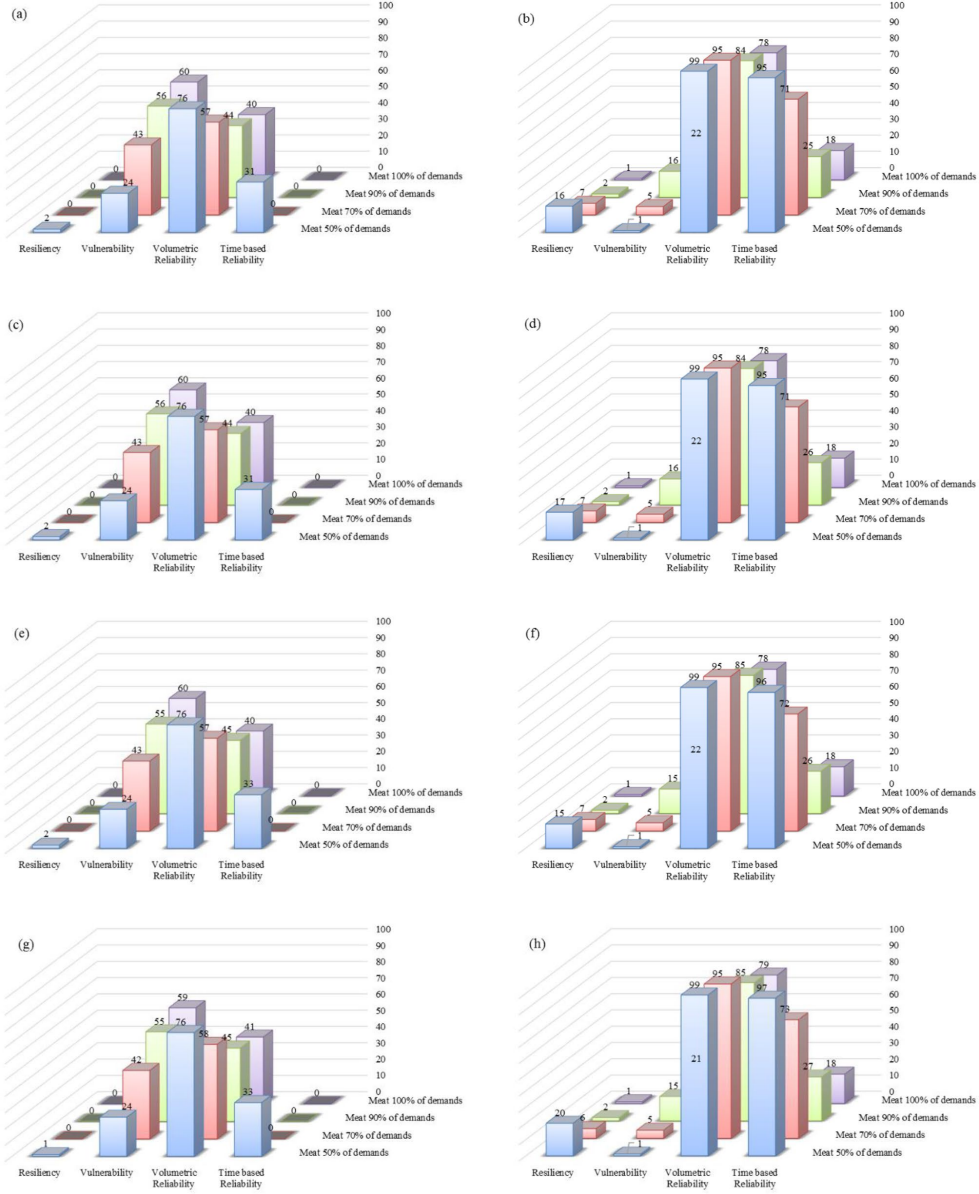

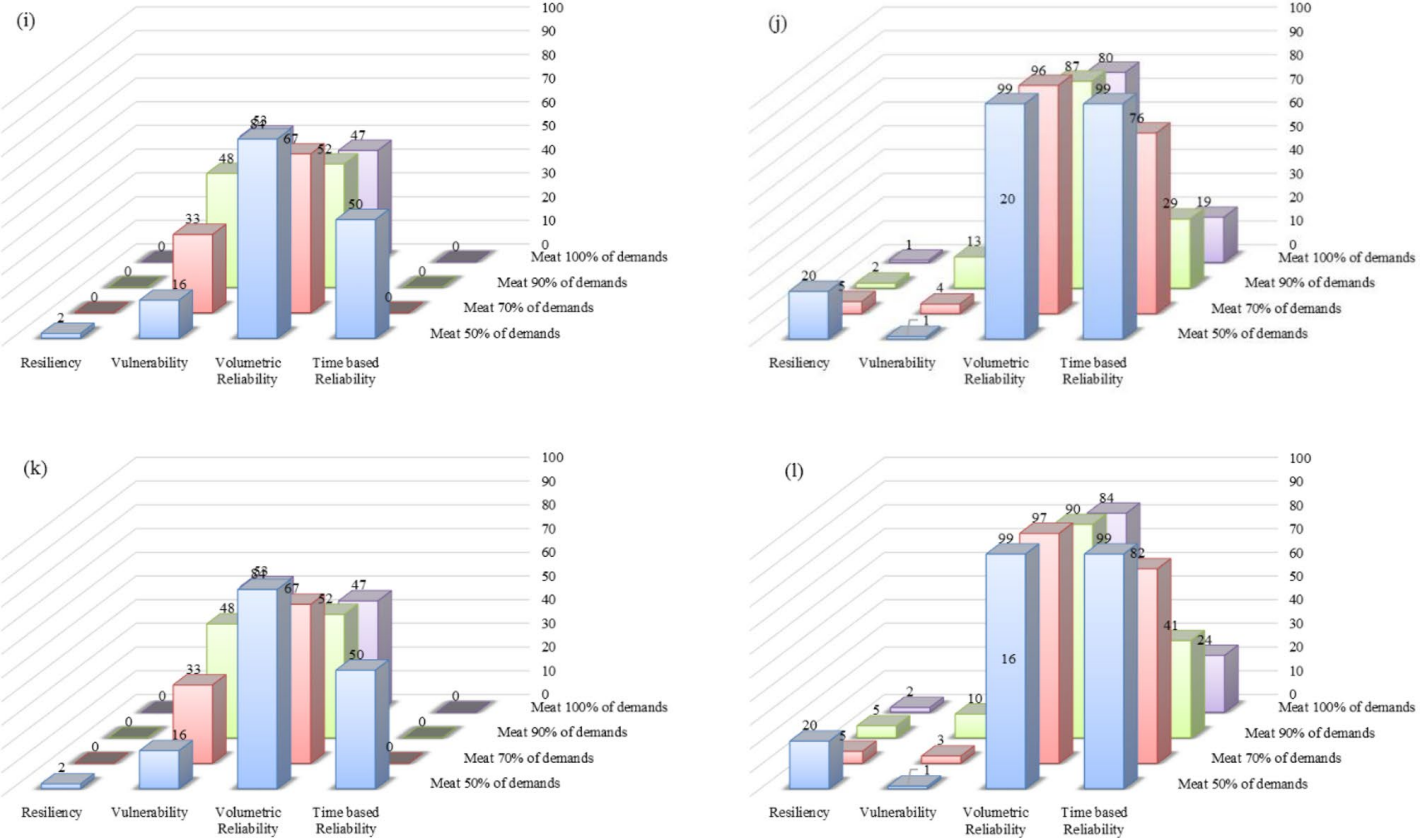
Performance criteria of the predicted lake volumes in future periods for Option 1 under six management scenarios: (**a**) management Scenario 1 in 2021–2050; (**b**) management Scenario 1 in 2051–2080; (**c**) management Scenario 2 in 2021–2050; (**d**) management Scenario 2 in 2051–2080; (**e**) management Scenario 3 in 2021–2050; (**f**) management Scenario 3 in 2051–2080; (**g**) management Scenario 4 in 2021–2050; (**h**) management Scenario 4 in 2051–2080; (**i**) management Scenario 5 in 2021–2050; (**j**) management Scenario 5 in 2051–2080; (**k**) management Scenario 6 in 2021–2050; and (**l**) management Scenario 6 in 2051–2080.

**Figure 11:**
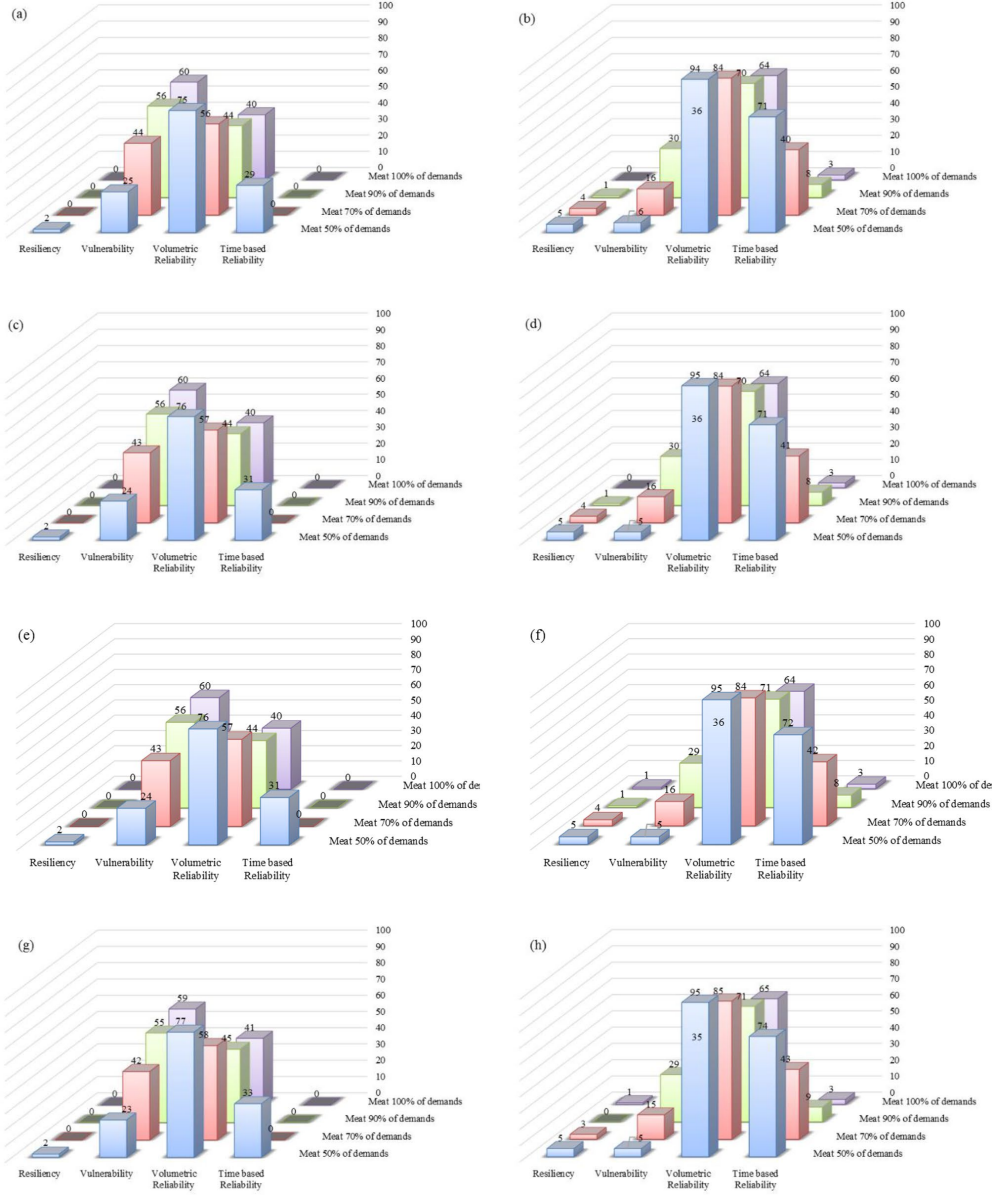

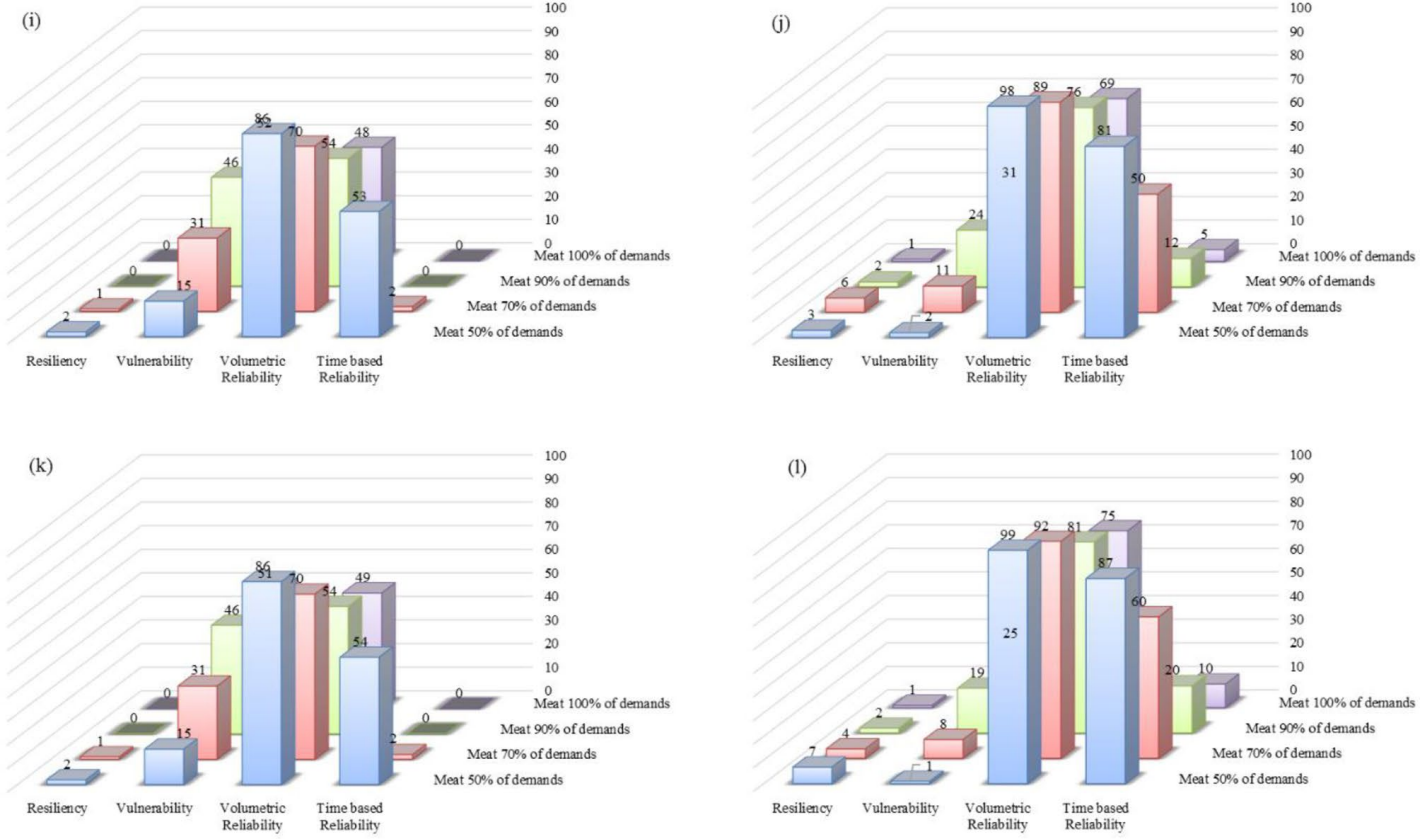
Performance criteria of the predicted lake volumes in future periods for Option 2 under six management scenarios: (**a**) management Scenario 1 in 2021–2050; (**b**) management Scenario 1 in 2051–2080; (**c**) management Scenario 2 in 2021–2050; (**d**) management Scenario 2 in 2051–2080; (**e**) management Scenario 3 in 2021–2050; (**f**) management Scenario 3 in 2051–2080; (**g**) management Scenario 4 in 2021–2050; (**h**) management Scenario 4 in 2051–2080; (**i**) management Scenario 5 in 2021–2050; (**j**) management Scenario 5 in 2051–2080; (**k**) management Scenario 6 in 2021–2050; and (**l**) management Scenario 6 in 2051–2080.

The effects of applying the six management scenarios for Option 1 (one-reservoir option) and Option 2 (six-reservoir option) were compared. Four performance criteria, i.e., time-based reliability, volumetric reliability, vulnerability, and resiliency for four performance thresholds (100%, 90%, 70%, and 50%) were selected to determine the most effective scenario and to assess the effectiveness of the Bukan reservoir. The assessment results under the six management scenarios for Options 1 and 2 are listed in Figs. 10 and 11 for the period of 2021–2050 and 2051–2080.

The performance criteria for the simulated volumes of Lake Urmia in the period of 2021–2050 corresponding to Option 2 are better than those for Option 1. This is an indication Option 2, in which all six reservoirs would be operational, is preferable so long as meeting agricultural demand is the main objective.

The time-based reliability of the lake water volume for Option 2, with a performance threshold of 70% in the first future period, increases from 0 in management Scenario 1 to 2% in management Scenario 6. This 2% increase in reliability is achieved when the agricultural water demand decreases by 100%, while the value of the time-based reliability of the lake water volume is constantly 0 for Option 1. The lake volumetric reliability increases by an average of 10% in Option 1 for the second future period under all six management scenarios if the performance threshold is reduced from 70 to 50%, while the lake time-based reliability increases by an average of 30% in Option 2.

The simulated lake water levels in 2021–2050 would be lower than those in the 2051–2080 period, which means the values of the four performance criteria for the simulated lake water volumes in the second future period would be better than those in the first future period when the same policy is implemented in both future periods. For instance, under Scenario 1 and Option 1 the vulnerability values decrease from 24%, 43%, 56%, and 60% in 2021–2050 (Fig. 10) to 1%, 5%, 16%, and 22% (Fig. 10) in 2051–2080 with respect to the performance thresholds of 50%, 70%, 90%, and 100%, respectively. Comparison of the management scenarios revealed that there was no significant difference in the performances corresponding to management Scenarios 1 through 4, while all performance criteria were improved in management Scenarios 5 and 6. Our results suggested that implementing stricter policies would help improve the conditions of the lake and help its ecosystem.

The Zarinerud sub-basin and Bukan reservoir play central roles on the Lake Urmia hydrology. Future lake studies should focus on the sub-basins and reservoirs that have govern the lake’s hydrology^8,50^. Moghadasi et al.^51^ showed that agricultural water use should be reduced by 25–35% in East Azerbaijan, and by 15–25% in West Azarbaijan to achieve optimal water allocations in the Lake Urmia basin.

## Conclusions

The temperature and precipitation data were extracted for the Lake Urmia basin from the HADCM3 projections under emission Scenario A2. These projections were downscaled with the delta-change method for future periods 2021–2050 and 2051–2080. The inflows to the Bukan reservoir and the downstream flows were simulated with the ANN model. The inflows of five other reservoirs and their downstream flows were calculated using the Bukan reservoir data and the ratios of the values of five reservoirs’ variables to the corresponding values for Bukan reservoir located in the Zarrineh sub-basin. The SD method was applied to simulate the water balance of the Lake Urmia basin in the future periods. Several management scenarios were developed to improve the condition of Urmia Lake. The following conclusions were obtained from this study:

- The temperature in the Lake Urmia basin is expected to rise in both future periods. The precipitation in region upstream of the Bukan reservoir is expected to decrease by 32% in the first future period (2021–2050) and increase by 0.4% in the second future period (2051–2080). The precipitation is expected to increase by 0.3% in the first period and decrease by 2.38% in the second period in the regions downstream of the Bukan reservoir.
- The lake water level in the first future period would be lower than its long-term average and the ecological water level.
- The values of the performance criteria of the predicted lake water volumes in the first future period would be lower than those in the second future period under all management scenarios. But the rate of increase in the performance criteria by decreasing the agricultural water allocation (changing from management Scenario 1 to management Scenario 6) in the first future period would be larger than that of the second future period.
- Lake Urmia would experience a considerable loss of water because of drought even if the agricultural water allocation is eliminated under some management scenarios.

More in-depth studies could help shed light on the intricacies and challenges of preserving Lake Urmia.

## Data Availability

The data that support the findings of this study are available from the corresponding author upon reasonable request.

## Code availability

The codes that support the findings of this study are available from the corresponding author upon reasonable request.
